# Feasibility and Utility of Telephone-Based Psychological Support for People with Brain Tumor: A Single-Case Experimental Study

**DOI:** 10.3389/fonc.2015.00071

**Published:** 2015-03-26

**Authors:** Stephanie Jones, Tamara Ownsworth, David H. K. Shum

**Affiliations:** ^1^School of Applied Psychology and Griffith Health Institute, Griffith University, Brisbane, QLD, Australia

**Keywords:** neuro-oncology, brain tumor, psychological distress, psychotherapy, telephone-based support, counseling

## Abstract

Rates of psychological distress are high following diagnosis and treatment of brain tumor. There can be multiple barriers to accessing psychological support, including physical and cognitive impairments and geographical limitations. Tele-based support could provide an effective and more flexible option for delivering psychological interventions. The present study aimed to investigate the feasibility and utility of a telephone-based psychotherapy intervention for people with brain tumor. A single-case multiple-baseline design was employed with a 4–7-week baseline phase, 10-week treatment phase, and 5-week maintenance phase including a booster session. Four participants with a benign or malignant brain tumor (three males and one female; aged 34–49 years), received 10 sessions of tele-based therapy and a booster session at 4 weeks post-treatment. Levels of depression, anxiety, and illness cognitions were monitored on a weekly basis throughout each phase whilst measures of quality of life, stress, and self-concept were administered at the start and end of each phase. Weekly measures were analyzed using a combination of both visual analysis and Tau-*U* statistics. Of the four participants, two of them demonstrated significant gains in mental health (depression and/or anxiety) and a significant decrease in their levels of helplessness (*p* < 0.05). The other two participants did not show gains in mental health or change in illness cognitions. All participants reported improvement in quality of life post-treatment. The results of the study provide preliminary support concerning the feasibility and utility of tele-based therapy for some people with brain tumor. Further research examining factors influencing the outcomes of tele-based psychological support is needed.

## Introduction

Levels of psychological distress following brain tumor diagnosis are high, with 41–47% of people found to experience depression or anxiety beyond the primary treatment phase (1). There are numerous practical barriers to people accessing face-to-face (FTF) psychological support, including cognitive and physical difficulties, costs, and geographical distance. Despite this, there is very limited research on the feasibility and efficacy of flexible delivery modes of psychological intervention (e.g., telephone, internet).

Depression has been found to be consistently related to poor quality of life for people with brain tumor (2–6). Some symptoms of depression are likely to arise directly from the biological effects of the tumor and its treatment (e.g., weight loss, sleep disturbance, concentration difficulties). Other symptoms may develop in reaction to the threat to life and stressors associated with functional impairments and activity restrictions (7). In particular, people with brain tumor experience a prominent sense of threat and uncertainty about the future (8). For example, people with low-grade glioma may live with relatively mild neurological symptoms for many years and be able to perform normal occupational activities until the disease progresses and their functional state declines. Conversely, people with a Grade IV glioma may not be able to resume occupational roles and they often face a much shorter life expectancy with rapid functional decline.

Adelbratt and Strang (9) identified a common theme of “death anxiety,” which referred to the preoccupation with threat to life experienced by the person with brain tumor and their next of kin. Symptoms and gradual loss of functions were seen as metaphors of dying and death (9). These illness appraisals can impact both physical and psychological health (10). Higher levels of psychological distress are generally associated with perceptions of high threat or helplessness and low levels of controllability or self-efficacy regarding coping (11). For example, people with higher perceptions of threat and lower perceptions of controllability 2 weeks after stroke had poorer psychological adjustment at 6 months post-stroke (12).

With the combined effects of brain injury and cancer, brain tumor poses some unique stressors. Cancer is often considered uncontrollable and highly threatening, with limited potential for benefit (10, 13, 14). Most people experience tumor re-growth or progression and functional decline. The physical, cognitive, and behavioral impairments associated with the tumor and its treatment lead to increased dependence on others, relationship strain, and inability to resume valued activities (e.g., driving and work). Experiencing a sense of threat and low controllability in combination with severe functional impairments can have a devastating impact on quality of life.

Antonovsky’s (15) sense of coherence (SOC) model has been applied to understand how people strive to maintain well-being in the context of adversity. The SOC model proposes that three components, namely, comprehensibility (understanding of what is happening), manageability (perceived ability to access resources to cope), and meaningfulness (capacity to find meaning within the situation) influence people’s psychological and physical well-being. In support of this model, stronger SOC has been found to be protective against the development of depression and anxiety in people with cancer and their partners (16). Further, there is evidence to suggest that SOC can be enhanced through intervention (17, 18).

Closely related to the concept of SOC, Salander et al. (19) found that people with malignant glioma varied in their “time of everyday life,” or level of engagement in activities that were similar to those prior to their diagnosis, and “time of disease” or extent to which they were occupied with the disease and its treatment. One-third of working age patients described a loss of life continuity, only experiencing “time of disease”; these patients reported an absence of everyday living. The remaining two-thirds of individuals were found to have spent a period focusing on “time of life” before they progressed to “time of disease.” “Time of life” included work, hobbies, and activities of daily living which serve to maintain a sense of connection to everyday life, and foster hope, not of a cure, but of a remaining life not dominated by disease and death.

Overall, this research highlights that interventions focusing on making sense of, finding meaning, and participation in meaningful “time of life” activities have the potential to enhance psychological adjustment and quality of life after brain tumor. Despite the well-recognized need for psychological support for people with brain tumor and their families, there is limited published research on the efficacy of interventions (4, 8, 20–22). Controlled trials of psychological interventions for people with cancer have typically excluded people with brain tumor or those with cognitive dysfunction (23–25), thus limiting the capacity to generalize findings from the general cancer literature.

A review of counseling and rehabilitation interventions for adults with brain tumor identified 13 studies, which included 6 case studies or case series (see Table 1), 4 RCTs, and 3 pre-post group studies with no control group (see Table 2). Overall, there was evidence of gains in psychological well-being from case studies. However, although the controlled trials of cognitive rehabilitation demonstrated some gains in cognitive functioning and strategy use (21, 26, 27), such gains did not extend to psychological well-being or quality of life. This suggests that rehabilitation focusing on cognitive impairments may not be sufficient to improve broader psychosocial well-being. Promisingly, a recent study protocol (28) described a study underway that is investigating the efficacy of internet-based guided self-help for people with glioma with mild to moderate depression.

**Table 1:** **Summary of case studies evaluating psychological interventions for people with brain tumor**.

Reference	Intervention	Tumor characteristics	*n*	Intervention outcomes
Rao and Bieliauskas (29)	Psychological (16 couple sessions) and cognitive retraining (16 sessions)	Grade II–III	1	Improvements in neuropsychological functioning and behavior (e.g., social interactions, leisure, driving skills), and efficiency on work tasks
Sherer et al. (30)	Cognitive and vocational rehabilitation in clinic and community setting	High grade	13	Gains in independence for six participants (six remained the same, one declined) and productivity for eight participants (four remained the same, one declined) which was maintained at 8-month follow-up
Kowal et al. (31)	Emotion-focused couples therapy (12 sessions)	Low grade	1	Description of positive psychological outcomes
Tepper (32)	Psychosocial support	High grade	4	Description of positive psychological outcomes
Duval et al. (33)	Cognitive and ecological rehabilitation (26 sessions), information meetings (two sessions)	Grade II	1	Improvement in working memory at 3-month follow-up with generalization to everyday life
Whiting et al. (34)	2-h session of psychoeducation, communication, and relaxation skills training	Grade II	1	Decrease in target behavior and increase in knowledge of strategy use

**Table 2:** **Summary of group studies evaluating psychological interventions for people with brain tumor**.

Reference	Intervention	Design and sample characteristics	*n*	Intervention outcomes
Locke et al. (21)	12 sessions of cognitive rehabilitation (CR) and problem-solving therapy vs. standard medical care	RCT; mixed grades	19	Positive feedback from people with brain tumor and caregivers on the program; 88% used compensation strategies and 88% found the intervention helpful. No significant differences on quality of life (QOL), functional capacity, mood, or fatigue between control and intervention group at 3-month follow-up
Gehring et al. (26)	CR (retraining and compensation, six sessions); 3-month telephone-based booster	RCT with waiting list; Grade II and III	140	Significant effects at post-treatment for subjective cognitive function and perceived burden; not maintained at 6-month follow-up. At 6-month follow-up, significant gains on tests of attention and verbal memory and improvements with mental fatigue
Hassler et al. (35)	10 sessions of group cognitive training (attention, verbal, and memory skills) over 12 weeks	Pilot study with no control group; Grade III and IV	11	Significant improvement in verbal memory at post-intervention
Zucchella et al. (27)	16 sessions of CR for 4 weeks	RCT; mixed grade	58	Significant improvement in cognitive functioning at post-intervention
Khan et al. (36)	Individualized social support program: interview plus peer support or community education/counseling	Prospective longitudinal pre-post design; mixed grade	43	Significant improvements in psychological functioning, physical QOL, coping strategies, functional, and cognitive independence at 6-week follow-up. Gains in anxiety, stress, and QOL were not maintained at 6-month follow-up, although broader psychosocial gains were maintained long-term
Ownsworth et al. (37)	10 sessions of home-based psychotherapy	RCT with wait list; mixed grade	50	Significantly reduced depression and improvements in existential well-being and QOL at post-intervention and 6-month follow-up

The first RCT of a psychotherapy intervention for people with brain tumor was conducted by Ownsworth and colleagues (37). The 10-session Making Sense of Brain Tumor (MSoBT) intervention (*n* = 50) was home-based, goal-directed, and guided by principles of the SOC model. Although the focus of support was mainly on the person with brain tumor, involvement of family members was strongly encouraged and 60% of programs involved a family member who attended 1–10 sessions. An evaluation of post-intervention outcomes identified that participants with brain tumor experienced significantly lower levels of depression and higher levels of existential well-being and global quality of life relative to wait list controls. At the 6-month follow-up, participants were found to have significantly better psychological well-being than prior to the intervention (37).

Despite the promising psychological outcomes of the FTF MSoBT intervention, the program was both time and cost intensive. The authors identified that to maximize participant engagement, practitioners often drove over an hour to provide an intervention for the person with brain tumor and his or her family members (37). Such travel time may not be feasible for service delivery within the community. Furthermore, due to travel time, the program was restricted to people living within a major metropolitan area. To reduce such barriers to accessing psychotherapy, tele-health may be an option for service provision.

Telephone-based and internet interventions are increasingly being utilized to reduce barriers to accessing psychotherapy. A meta-analysis by Andersson and Cuijpers (38) for internet and other computerized psychological treatments for depression found a moderate to large average effect size (*d* = 0.41) across 12 studies. The authors noted that the studies that provided some form of direct therapist support to participants (e.g., email, telephone contact, or additional FTF contact) yielded larger effect sizes (*d* = 0.61). Hammond and colleagues (39) compared the clinical and cost-effectiveness of FTF and over-the-telephone (OTT) low-intensity CBT interventions for mild to moderate anxiety and depression (*n* = 4,106). They found that outcomes of the OTT and FTF interventions were comparable, with the exception of those with more severe illness, where FTF was found to be more effective. The service costs of OTT were found to be approximately one-third lower than FTF sessions (39). The researchers proposed that OTT interventions are a convenient and effective mode of delivery for those requiring low-intensity interventions (39).

From a practical perspective, tele-based therapy may result in lower attrition and greater access to psychological therapy for people restricted by mobility or geography. In a meta-analysis of FTF therapies between 2000 and 2010, Swift and Greenberg (40) reported mean attrition rates of 19.7%. In contrast, Mohr and colleagues (41) found that the mean attrition rate across 12 RCTs of tele-based psychotherapy for depression was only 7.6%. Tele-based psychotherapy has been shown to be effective for improving emotional adjustment for people with multiple sclerosis (42), HIV-AIDS (43), depression (44), and traumatic brain injury (45). Such findings support the potential utility of telephone-based psychotherapy for people with a brain injury.

Whilst tele-based therapy may increase the opportunity for people with brain tumor to access psychotherapy, neuropsychological impairments such as language difficulties, memory problems, distractibility, and difficulties with sustained attention may pose a barrier to engagement and efficacy for treating mood problems. Psychotherapy relies upon on verbal communication, including understanding of spoken and written information. Tele-based therapy does not allow for the use of visual aids and techniques (such as diagrams or handouts) to support understanding of concepts. Instead, there is reliance on auditory communication in terms of both verbal and non-verbal responses (e.g., sighs, crying, and laughing), which may affect the therapeutic alliance (46). Telephone-based therapy also has the increased potential for interruptions and distractions, particularly when conducted in the person’s home. In addition, family members may have more difficulty engaging in the therapeutic process. Therefore, an evaluation of the feasibility and utility of telephone-based therapy for people with brain tumor is clearly warranted.

Accordingly, the broad aim of the present study was to evaluate the feasibility and utility of a telephone-based psychotherapy intervention for people with brain tumor. The present study seeks to extend on the previous FTF MSoBT intervention for people with brain tumor (37). An additional booster session was included 2 weeks after the 10-session program to support maintenance and generalization of gains (47). In relation to utility, it was hypothesized that telephone-based psychotherapy would result in a significant decrease in levels of depression, anxiety, and hopelessness between the baseline and treatment phase (as measured weekly), which would be maintained at 5 weeks follow-up. Additionally, it was hypothesized that there would be a significant increase in levels of acceptance and perceived benefit between the baseline and treatment phase (as measured weekly), and that these gains would be maintained at 5 weeks follow-up. Broader gains in quality of life and self-concept were also assessed.

## Materials and Methods

### Design

**Figure 1:**
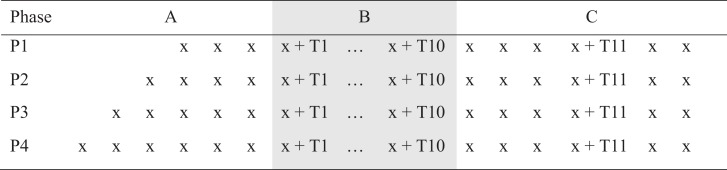
**Multiple baselines across participants design [A, baseline; B, treatment; and C, maintenance and booster (T11) session]**.

A single-case experimental design (SCED) with multiple baselines across participants (see Figure 1) was used to examine the impact of a telephone-based therapeutic intervention on psychological well-being. Single-case methodology is beneficial when evaluating a new treatment in conditions that are rare, in which it is difficult to obtain large samples with homogenous characteristics. The design entails repeated measurements of functioning over time to evaluate the impact of treatment relative to the baseline period (48).

Multiple-baseline designs reduce the likelihood of extraneous, potentially confounding factors influencing the results (49, 50). Beeson and Robey (51) recommended a minimum baseline of three data points to control for threats to validity. Utilizing repeated observation prior to the commencement of the intervention allows for analysis of trends in the data both within and between phases. In the present study, there was a minimum of four baseline data points (random allocation P1) and a maximum of seven baseline data points (random allocation P4), prior to the 10 treatment sessions. Participants were randomly allocated to baseline length (four, five, six, or seven) using a pre-determined randomized computer sequence with concealed allocation of numbers placed in sealed opaque envelopes (50).

### Participants

In the earlier MSoBT program [see Ref. (37)], individuals with primary brain tumor were recruited through major hospitals, neurosurgery clinics, and community services supporting people with cancer and brain injury. When the FTF MSoBT program ceased recruitment, participants inquiring about the initial program after July 2012 were referred to the telephone-based program. Adults with a primary brain tumor were eligible to participate in the study, irrespective of their tumor type and status. Participants were eligible from across Queensland. Participants undertaking current psychological interventions related to the effects of their brain tumor were not eligible to participate. Very severe cognitive deficits or receptive and/or expressive language deficits were considered likely to preclude telephone-based assessment or therapy. A telephone-based cognitive assessment tool was used to screen for cognitive and language deficits to determine eligibility. Additional eligibility criteria included ongoing access to a telephone, availability for weekly telephone assessment and therapy over a 20–24-week period (inclusive of baseline, treatment, and maintenance phases), and no significant hearing deficits that would preclude the use of a telephone.

A sample of four participants was considered an appropriate number for a SCED with the length of baseline varying from 4 to 7 weeks. The demographic and medical characteristics of the four participants (Mark, John, Robyn, and Samuel) are summarized in Table 3. More details of the health, cognitive, and psychological status of each participant is provided in the Section “Results.”

**Table 3:** **Summary of participants’ demographic and health characteristics**.

Characteristics	Mark^a^ (P1)	John^a^ (P2)	Robyn^a^ (P3)	Samuel^a^ (P4)
Age (years)	43	34	49	40
Gender	Male	Male	Female	Male
Highest level of education	Post-secondary school diploma	Secondary (high school)	Undergraduate degree	Undergraduate degree
Current employment	Part-time	Part-time	Full-time	Full-time
Current relationship status	Divorced, no children	Married, three children	Divorced, two children	Single, no children
Time since diagnosis	13 years	2.5 years	3 months	16 years
Brain tumor type	Cystic astrocytoma	Anaplastic astrocytoma	Pituitary tumor	Oligoastrocytoma
Tumor malignancy	Grade I	Grade III	Grade I	Grade II
Brain tumor location	Hypothalamus/optic pathway	Left temporal lobe	Pituitary gland	Left temporal lobe
Treatment/s	Surgery	Surgery	Surgery	Surgery
	Radiotherapy	Radiotherapy	Hormone replacement therapy	Radiotherapy
		chemotherapy		chemotherapy
		Anti-convulsants		Anti-convulsants
Geographical location	Regional	Regional	Metropolitan	Metropolitan
Ability to drive	Yes	Yes	No	No

*^a^Pseudonym used to protect participant’s identity*.

### Measures

#### Cognitive screening

In the initial telephone session, participants completed the brief test of adult cognition by telephone [BTACT; (52)] to screen for very severe cognitive deficits that were considered likely to affect people’s capacity to engage in the intervention program. The BTACT is a brief (20 min) test of auditory attention, processing speed, memory, verbal fluency, and reasoning. In this study, five of the seven subtests were completed as follows: Word List Recall, Digits Backward, Category Fluency, Backwards Counting, and Short-Delay Recall. The BTACT has sound psychometric properties and has been validated in the general population (*n* = 4268).

Results on the BTACT indicated that Samuel performed in the “below average” range relative to age norms on measures of immediate and delayed memory, verbal fluency, and processing speed. Robyn’s scores indicated “below average” performance on a delayed verbal memory task and Mark’s scores indicated “below average” performance on a verbal fluency task. John’s performance was in the “average” range for all five domains. Although Samuel demonstrated age-related impairments on four cognitive tasks (i.e., >1 to <2 SD below the norms), he was considered to have adequate cognitive functioning to undertake a telephone-based therapy program.

#### Psychological outcomes

At the start and end of each phase, participants completed the full set of self-report measures, including the 21 item Depression, Anxiety, and Stress Scale [DASS-21; (53)], Generalized Anxiety Disorder Scale [GAD-7; (54)], FACT-Brain (55), Illness Cognition Questionnaire [ICQ; (10)], and Continuity and Discontinuity of Self Scale [CDSS; (56)]. The brief set of outcome measures for session-by-session assessment included the depression scale of the DASS-21, GAD-7, and ICQ. The Session Rating Scale [SRS; (57)] was completed after every therapy session (from session two onward) to assess therapeutic alliance.

##### Mood state

The seven-item depression subscale of the DASS-21 was designed to assess symptoms of depression and has been validated for use with people with brain tumor (7). The clinical cut-offs are: normal, ≤9; mild, 10–13; moderate, 14–20; severe, 21–27; and extremely severe, ≥28. The GAD-7 is a measure of symptoms of generalized anxiety disorder which has been validated in primary care settings (54) and the general population (58). The clinical cut-offs for the scale are: normal, <5; mild, 5–9; moderate, 10–14; and severe, ≥15.

##### Illness cognitions

The ICQ is an 18-item measure of illness cognitions for people with chronic disease (10). It was modified in this study so that items applied to brain tumor (i.e., the word “illness” was replaced with “tumor”). The subscales measure helplessness (e.g., *“Because of my tumor, I miss the things I like to do most*”), acceptance (e.g., “*I can handle problems related to my tumor*”), and perceived benefits (e.g., “*Dealing with my tumor has made me a stronger person*”). Higher scores indicate increased levels of helplessness, acceptance, or perceived benefits.

##### Quality of life

The FACT-G (33 items) is comprised of four subscales that assess physical, social/family, emotional, and functional well-being aspects of health-related quality of life (55). An additional subscale developed by Weitzner and colleagues (59) assesses brain-related concerns. Higher scores indicate increased quality of life, with scores ≥0.5 SD below the norms (*M* = 80.1, SD = 18.1) indicating low quality of life (55).

##### Self-concept

The CDSS (24 items) assesses discontinuity of self (e.g., “*I sometimes give up on something because it is too much trouble*”), continuity of self (e.g., “*I have control of my life*”), and continuity with others (e.g., “*I feel accepted by others*”). Higher scores indicate greater levels of discontinuity of self (range = 1–36) or continuity of self (range = 1–15) and continuity with others (range = 0–21). Originally developed for the stroke population, the CDSS was considered suitable for use with people with brain tumor based on research indicating that sense of self and life continuity can often be disrupted (19).

##### Therapeutic alliance

The SRS, Version Three (57) was converted to an 11-point scale (e.g., 0,“I did not feel heard, understood, and respected” to 10,“I felt heard, understood, and respected”) to assess: (1) quality of the relational bond, (2) agreement between the individual and therapist regarding the goals, and (3) agreement on the method and approach used.

### Procedure

Ethical clearance was obtained from the Griffith University Human Research Ethics Committee (PSY/37/10/HREC) as part of the larger MSoBT project. During the initial screening process, demographic and medical information was obtained from participants (e.g., tumor type, time since diagnosis, age, gender, and employment status). Participants were provided with details about the study OTT and through written information posted to them. Participants returned a signed consent form prior to commencing the study.

Participants were randomly allocated to one of the four baselines lengths. Numbers (4–7) corresponding to the length of baseline were placed in sealed opaque envelopes and then randomly ordered. The envelopes were opened in consecutive order as each participant entered the study. Prior to commencing the initial assessment, participants were posted a booklet of the outcome measures for ease of administration. The booklet also contained a copy of the consent form, SRS, and plastic sleeves for participants to store any notes they made. The booklet was divided into sections for ease of use.

**Figure 2:**
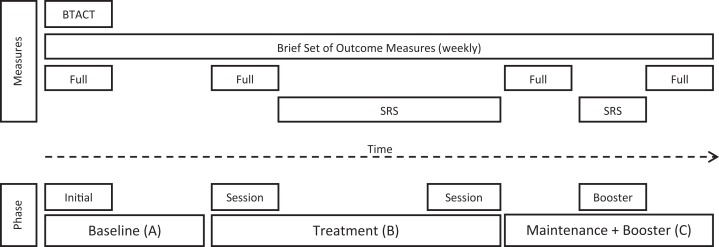
**Phases of the intervention program and assessment time points (SRS, Session Rating Scale)**.

After allocation, the researcher conducted the initial assessment session, which included the BTACT and full set of outcome measures. Participants were assessed on a weekly basis on a brief set of outcome measures (“brief”) for the duration of their allocated baseline (see Figure 2). Prior to the commencement of therapy, participants were re-administered the full set of outcome measures, with the exception of the BTACT. During therapy, participants completed the brief set of outcome measures at the start of the telephone call, just prior to therapy. Participants subsequently completed 10 sessions of individual telephone-based therapy with an additional booster session 4 weeks after their completion of the 10 sessions.

The SRS was completed after each telephone-based therapy session from session two onward, including the booster session. The full set of outcome measures was re-administered after the completion of the 10 telephone-based therapy sessions and again at the end of the maintenance phase, 6 weeks later, as seen in Figure 2.

### Therapeutic intervention

Consistent with the FTF program, 1 h telephone therapy sessions comprised of both core (sessions 1, 2, and 10) and individualized components, with the latter tailored to each participant’s specific therapy goals and life circumstances. During the initial session, participants described their experience of symptom onset, diagnosis, treatment, and the impact of the tumor and its treatment on daily living (i.e., “telling my story”). Session two explored personal values and associated goals and priorities. From the information gained and rapport built during sessions one and two, three to five therapy goals were collaboratively set. Goals most typically related to understanding the effects of the brain tumor, learning strategies to manage negative emotions and cognitive difficulties, improving relationships, and increasing social participation and healthy lifestyle behaviors.

The tenth session summarized the main content of prior therapy sessions and involved reflecting on gains and progress. A plan for maintaining skills and managing set-backs was also a focus of the session. The booster session also focused on maintenance of strategy use and skills generalization and discussed issues associated with termination of therapy.

Individual treatment modules included: psychoeducation on the brain and brain tumor, cognitive rehabilitation and associated strategies (e.g., memory and organization), cognitive-behavior therapy, psychoeducation on emotional and behavioral changes (e.g., symptoms of anxiety, depression, and panic attacks), mindfulness techniques (e.g., mindful eating, present focused awareness), pleasant activity scheduling, relaxation techniques (e.g., progressive muscle relaxation, abdominal breathing), couple and family support (communication, problem-solving), and existential and end-of-life discussions (i.e., family care plan).

### Data analysis

The analysis of the weekly repeated measures was conducted via a combination of visual inspection, and a Tau-*U* tool [singlecaseresearch.org; (60, 61)]. Steps to data analysis for the weekly measures included: checking relevant assumptions for SCED, analysis of baseline stability, and case-level analysis, including evaluation of treatment effects within phase. Data on broader subjective well-being measures was not subject to statistical analysis due to insufficient data points.

The Tau-*U* is a statistical approach derived from the Kendall Rank Correlation and Mann–Whitney-*U* tests, providing a combined index of non-overlapping data between two conditions (phases) and examination of trends both within and across phases. This type of analysis has been recommended for simple AB designs with particular strengths in controlling for baseline trend and variability, ceiling and floor effects, and has sensitivity to phase change when data have been collected over a short period of time, irrespective of baseline length (60). The Tau-*U* allows for analysis of baseline stability (A) and controls for trend. The analysis provides a more accurate evaluation of non-overlap or “dominance” of one phase over another (AB) than mean or median differences. The Tau-*U* has been found to have good statistical power for short data series and is robust to outliers or extreme scores (60). Tau-*U* is also relatively resistant to the effects of autocorrelation or serially correlated residuals, as demonstrated through field testing of 382 published data series, comparing the results before and after cleansing for autocorrelation (60).

Visual analysis allows for inspection as to whether there has been an observable change on the dependent variable by an intervention (62, 63). This method was used in conjunction with Tau-*U*, clinical cut-offs, and normative data.

## Results

### Analysis of baseline stability

**Figure 3:**
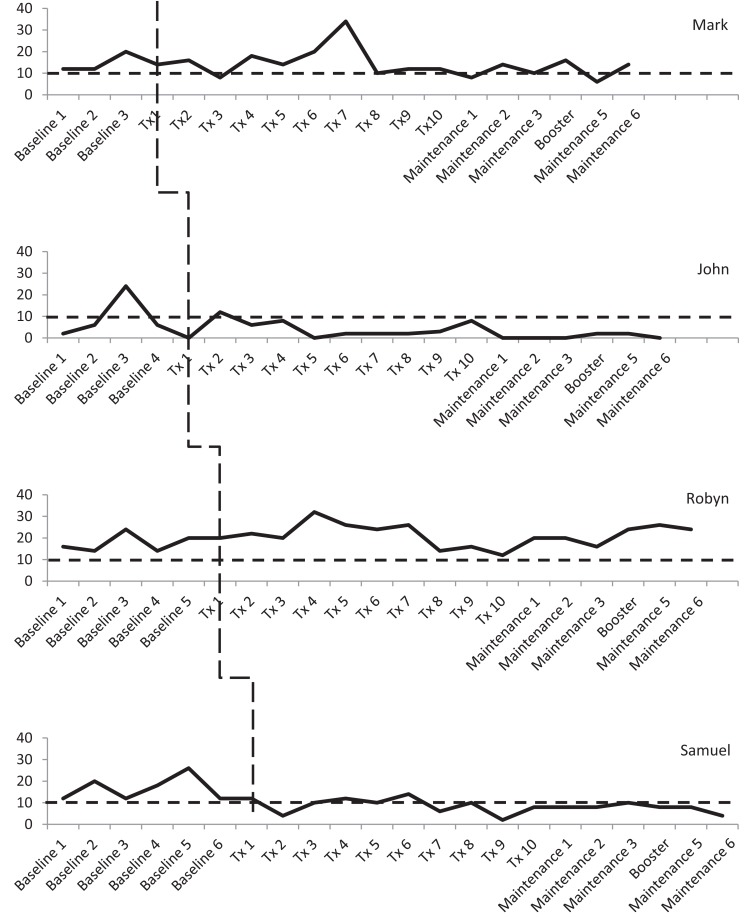
**Depression (DASS-21) levels across the three phases, with clinical cut-off for “mild” range (as indicated by broken line)**.

**Figure 4:**
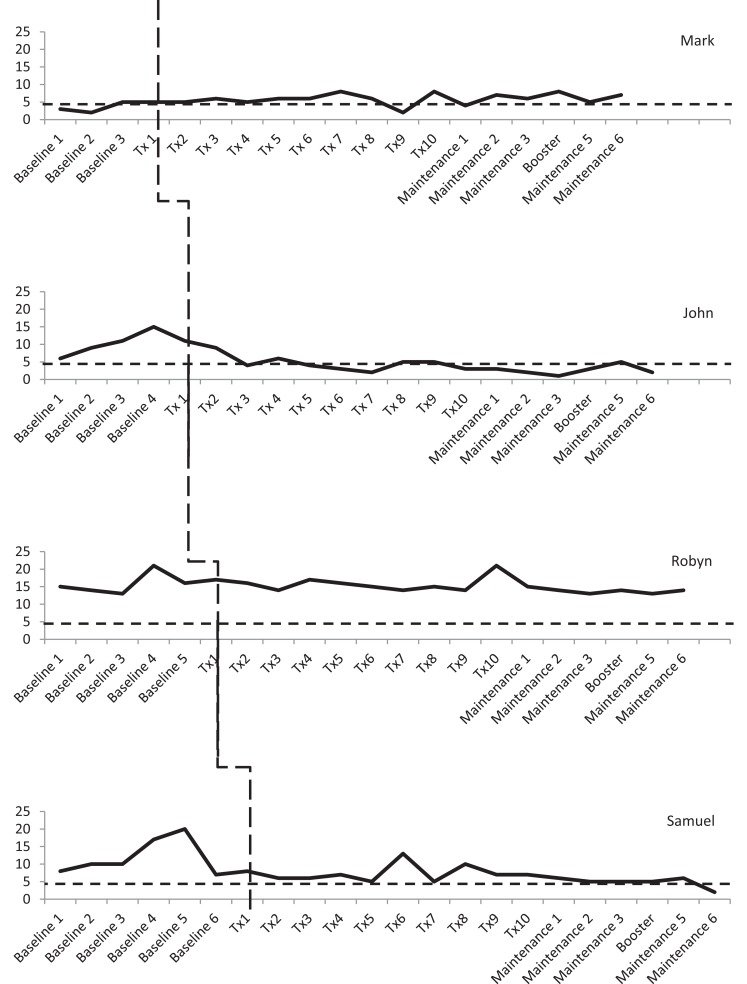
**Anxiety (GAD-7) levels across the three phases, with clinical cut-off for “mild” range (broken lines)**.

**Figure 5:**
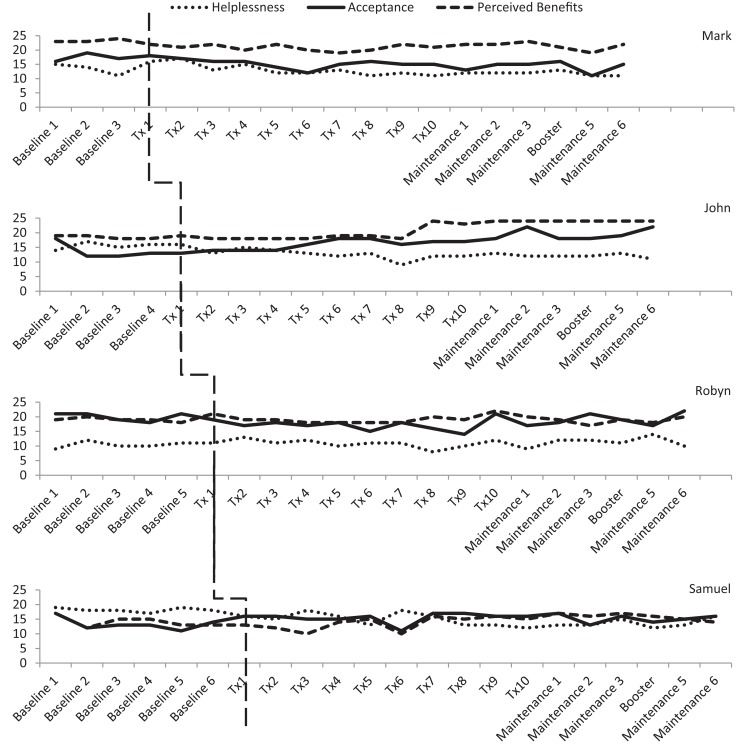
**Illness cognition levels on the Illness Cognition Questionnaire (ICQ) across the three phases**.

Three participants consistently scored within the clinical range for depression during the baseline phase, albeit there was some variability. As shown in Figure 3, Mark and Robyn’s scores varied between “moderate” and “severe” levels whilst John and Samuel’s scores ranged from “normal” to “severe.” There was also variability in anxiety scores for all four participants (see Figure 4). Mark’s scores ranged between the “normal” and “mild” range. John and Samuel’s scores varied between “mild” and “severe” levels of anxiety, whilst Robyn’s scores were in the “moderate” to “severe” range during the baseline phase. Three participants had scores consistently within the clinical range for anxiety during the baseline phase. Visual inspection of the ICQ data in Figure 5 indicated most variability on the helplessness scale for Mark and on the acceptance scale for John and Samuel. Robyn’s scores on the three ICQ scales appeared relatively stable.

### Case descriptions and evaluation of treatment effects

#### Mark

Mark had been diagnosed with a Grade I cystic astrocytoma near the hypothalamus 13 years ago. He was diagnosed after undergoing a routine pre-employment medical assessment overseas, which identified visual difficulties. He was told that he did not have long to live and was advised against further medical treatment. After further research into treatment, Mark underwent radiotherapy, which reduced the size of the tumor, and he subsequently had a partial resection. Since diagnosis, Mark reported a change in his personality and anger outbursts. His marriage broke down during the earlier years after his diagnosis and he has since had difficulty making friends and forming relationships. He reported some strained relationships with his family and a major loss when his mother died. He also reported ongoing difficulties with balance and strength (impacting on recreational activities) and a skin condition that affects his self-esteem and confidence. Mark was referred to the program by a family member who was concerned about how he was coping.

An analysis of the baseline phase identified no significant trend in DASS depression levels (Tau-*U* = 0.5, *p* = 0.308). A comparison of between phase variability (AB) indicated no significant difference between the baseline and treatment phases (Tau-*U* = −0.1, *p* = 0.777). Mark’s scores were consistently in the clinical range for depression (“mild” to “moderate”) with a notable increase between baseline assessments two and three (see Figure 3). During treatment, depressive symptoms were reduced between sessions two and three, with a subsequent increase in symptoms from sessions four to seven (i.e., “extremely severe” range). After session eight, his depression levels reduced to the “mild” range, until the end of treatment. During the maintenance phase, Mark’s depression scores varied between the “normal” and “moderate” ranges.

There was no significant trend in Mark’s GAD-7 anxiety levels in the baseline phase (Tau-*U* = 0.5, *p* = 0.308). Phase comparison (AB) indicated no significant difference between baseline and treatment phases (Tau-*U* = 0.625, *p* = 0.077). During the baseline, treatment, and maintenance phases, Mark’s anxiety levels ranged between “normal” and “mild” levels (see Figure 4).

On the ICQ, there was no significant trend in Mark’s baseline phase for levels of helplessness (Tau-*U* = 0, *p* = 1), acceptance (Tau-*U* = 0.333, *p* = 0.497), or perceived benefit (Tau-*U* = −0.167, *p* = 0.734). Phase comparison for helplessness identified no significant difference between baseline and treatment phases (Tau-*U* = −0.325, *p* = 0.358). Yet, over the course of treatment and maintenance phases, a gradual reduction in helplessness was observed (see Figure 5). Phase comparisons for levels of acceptance and perceived benefit were found to be significant between the baseline and treatment phases (Tau-*U* = −0.85, *p* = 0.016; Tau-*U* = −0.9, *p* = 0.011). Contrary to the hypothesis, Mark’s level of acceptance and perception of benefits associated with his brain tumor declined during the treatment phase.

On the measures of broader subjective well-being, Mark’s scores on the FACT-G improved during the baseline period (as shown in Table 4). This improvement mainly occurred on the social/family and functional well-being subscales. His initial baseline FACT-G score suggested low quality of life relative to the norms, which improved to within the “average” range for the normal population. On completion of the program, Mark’s scores remained in the normal range. There was a small improvement in self-concept (i.e., increase in continuity with others and decreased discontinuity of self).

**Table 4:** **Broader subjective well-being scores for Mark across phases**.

Measure	Initial baseline (A)	Final baseline (A)	End of treatment (B)	End of maintenance (C)
FACT-Brain
Physical	24	25	24	23
Social/family	11	15	17.5	14
Emotional	18	18	17	21
Functional	13	16	14	16
G	66	74	72.5	72
Brain	33	34	34	35
CDSS
Discontinuity self	34	33	33	31
Continuity self	13	11	15	12
Continuity others	19	18	20	21

Mark reported high levels of therapeutic alliance on the SRS. For all sessions, Mark consistently rated the alliance (relationship, goals and topics, approach or method, and overall) at the maximum score (10).

Overall, visual and statistical analysis of Mark’s psychological functioning indicated limited therapeutic benefits in terms of reducing his levels of depression and anxiety. Minor improvement in helplessness was found, although this coincided with decreased acceptance and perceived benefits. Minor improvements in social and functional well-being and self-concept were also reported.

### John

John was diagnosed with a Grade III anaplastic astrocytoma after a grand-mal seizure 2 years prior to the program. He reported that the seizure and diagnosis of brain tumor was unexpected and sudden, with no history of illness or need for medical attention. John underwent immediate debulking surgery, with subsequent rounds of chemotherapy and radiotherapy. He reported very little recollection of these events. Following treatment, John reported infrequent seizures and ongoing concerns about his limited memory of the diagnosis and events since the diagnosis. His main concern was that his brain tumor would preclude him from taking part in everyday activities and that those around him would treat him differently. John was also distressed about being a burden on his family and felt guilty that he needed rest breaks during the day. He also expressed grief that he would be unable to see his children grow old and achieve milestones (e.g., school graduations, birthdays, and weddings).

There was no significant trend in John’s DASS depression scores in the baseline phase (Tau-*U* = −0.1, *p* = 0.807). A comparison of between phase variability (AB) indicated no significant difference between the baseline and treatment phases (Tau-*U* = −0.1, *p* = 0.760). Visual inspection of the treatment phase identified that after the initial treatment session of “telling my story,” depression scores were in the “mild” range with scores reducing to the “normal” range for the remainder of the treatment phase and the maintenance phase.

No significant trend in GAD-7 anxiety levels was found in the baseline phase (Tau-*U* = 0.7, *p* = 0.086). Phase comparison indicated a significant reduction in anxiety between the baseline phase and treatment phase (Tau-*U* = −0.92, *p* = 0.005). Baseline scores ranged between “mild” and “severe” whilst scores in the treatment phase were in the “normal” to “mild” range. During the maintenance phase, John’s level of anxiety was in the “normal” range with the exception of maintenance session five, for which his score was in the “mild” range.

On the ICQ, there was no significant trend in the baseline phase for John’s levels of helplessness (Tau-*U* = 0.3, *p* = 0.462), acceptance (Tau-*U* = 0.0, *p* = 1), or perceived benefit (Tau-*U* = −0.2, *p* = 0.624). Phase comparison indicated a significant difference in illness cognitions between the baseline and treatment phases, which reflected a decrease in level of helplessness (Tau-*U* = −0.92, *p* = 0.005) and an increase in acceptance (Tau-*U* = 0.66, *p* = 0.043). Phase comparisons for perceived benefits indicated no significant difference between baseline and treatment phases (Tau-*U* = 0.08, *p* = 0.807).

Between the initial baseline assessment and final baseline assessment, John’s scores declined on the FACT-G (see Table 5), indicating low quality of life relative to the norms. However, on completion of the program, John’s scores had markedly increased from 72 to 98. At the end of treatment and maintenance phases, John reported minor improvements in self-concept (i.e., increased continuity of self, increased continuity with others, and decreased discontinuity of self).

**Table 5:** **Broader subjective well-being scores for John across phases**.

Measure	Initial baseline (A)	Final baseline (A)	End of treatment (B)	End of maintenance (C)
FACT-Brain
Physical	17	14	16	23
Social/family	20	20	21	25
Emotional	16	15	17	23
Functional	20	17	18	27
G	73	66	72	98
Brain	37	36	49	34
CDSS
Discontinuity self	28	28	28	23
Continuity self	11	5	13	15
Continuity others	18	21	21	21

John reported high levels of therapeutic alliance on the SRS throughout treatment [relationship (*M* = 8.8, SD = 0.91), goals and topics (*M* = 9, SD = 0.66), approach or method (*M* = 9, SD = 0.66), and overall (*M* = 9.2, SD = 0.78)].

In summary, visual and statistical analysis of John’s psychological functioning indicated a significant reduction in levels of anxiety and helplessness and increased levels of acceptance. There were no significant changes in John’s levels of depression or perceived benefits. Improvements in self-concept and quality of life were also observed.

### Robyn

Robyn reported a gradual onset of symptoms, including visual difficulties, facial numbness, right-sided numbness, lower limb edema, abnormal menstruation, emotional blunting, and significant weight gain. After 12 months of symptoms, Robyn was diagnosed with a benign (Grade I) pituitary tumor near the optic nerve. She had key-hole surgery 3 days after her initial neurosurgical consultation. On entry to the program 3 months post-diagnosis, Robyn reported difficulty coping with returning to full-time employment and part-time study. She reported concerns that her illness was invisible to others (i.e., family, friends, and colleagues) as there was no physical injury or sign of surgery. She also found it difficult to cope with heightened emotionality as prior to the surgery she had experienced emotional blunting. She reported being unhappy in her job, having a limited support system, and strained relationships with family members.

No significant trend was found in DASS-21 depression scores across the baseline phase (Tau-*U* = 0.2, *p* = 0.573). A comparison of between phase variability (AB) indicated no significant difference between the baseline and treatment phases (Tau-*U* = 0.333, *p* = 0.278). Robyn’s scores were consistently in the clinical range for depression, with scores mainly between the “moderate” and “severe” range, although there was a notable increase to an “extremely severe” level of symptoms after the third treatment session.

There was no significant trend in GAD-7 anxiety scores in the baseline phase (Tau-*U* = 0.333, *p* = 0.348). Phase comparison indicated no significant difference between the baseline and treatment phases (Tau-*U* = −0.033, *p* = 0.914). Visual inspection identified scores consistently between the “moderate” and “severe” range across all phases.

On the ICQ, there was no significant trend in the baseline phase for Robyn’s levels of helplessness (Tau-*U* = 0.333, *p* = 0.348), acceptance (Tau-*U* = 0.333, *p* = 348), or perceived benefit (Tau-*U* = 0, *p* = 1). Phase comparison for helplessness and perceived benefits indicated no significant difference between the baseline and treatment phases (Tau-*U* = 0.118, *p* = 0.704; Tau-*U* = −0.183, *p* = 0.551). Phase comparison for acceptance revealed a significant decrease in Robyn’s level of acceptance between baseline and treatment phases (Tau-*U* = −0.8, *p* = 0.009).

Between the initial baseline assessment and final baseline assessment, Robyn’s scores declined on the FACT-G (see Table 6), this decline occurred across all subscales, suggesting low quality of life relative to the norms. However, on completion of the program, Robyn’s scores had increased, with further improvements at the end of the maintenance period, reflecting improved quality of life. Between the initial and final baseline assessments, Robyn’s scores showed slight decline in self-concept (i.e., continuity with others, discontinuity of self). At the end of the maintenance phase, Robyn’s scores reflected improvements in self-concept (i.e., increased continuity with others and a return to initial baseline levels of discontinuity of self).

**Table 6:** **Broader subjective well-being scores for Robyn across phases**.

Measure	Initial baseline (A)	Final baseline (A)	End of treatment (B)	End of maintenance (C)
FACT-Brain
Physical	15	12	17	20
Social/family	18	15	17	17
Emotional	15	14	9	14
Functional	15	17	19	20
G	63	58	62	71
Brain	52	60	60	63
CDSS
Discontinuity self	24	32	26	27
Continuity self	13	12	13	13
Continuity others	18	16	17	21

Robyn reported high levels of therapeutic alliance on the SRS. With the exception of session seven (relationship score of 7), Robyn rated the alliance at a score of 8 or higher [relationship (*M* = 8.8, SD = 0.63), goals and topics (*M* = 8.9, SD = 0.31), approach or method (*M* = 9, SD = 0), and overall (*M* = 8.8, SD = 0.42)].

Overall, visual and statistical analysis of Robyn’s self-reported functioning indicated limited therapeutic benefits in terms of her levels of anxiety, depression, helplessness, and perception of benefits. Her level of acceptance of her illness actually declined across the program. Despite this, Robyn’s quality of life and self-concept increased throughout the intervention.

### Samuel

At the time of the intervention, Samuel was a single male working full-time as a shift-worker. He was in his twenties when he suffered his first grand-mal seizure, having had no previous history of seizures or major health concerns. He was diagnosed with a low-grade (II) oligoastrocytoma in the left temporal lobe and underwent surgical debulking, chemotherapy, radiation therapy, and ongoing use of anti-convulsants. He continued to have absence seizures at least twice per week. Samuel reported frequent panic attacks (more than one per week) and concerns about his memory and level of independence. He had been advised that the rate of tumor progression was unpredictable but was likely to recur (possibly at a higher grade), which contributed to his anxiety. In addition, because of the loss of his driver’s license, Samuel felt he was becoming a burden on his family and friends. Samuel reported strong family and social relationships and stable employment with a supportive employer.

There was no significant trend in DASS-21 depression scores during the baseline phase (Tau-*U* = −0.048, *p* = 0.881). A comparison of between phase variability (AB) indicated a significant reduction in level of depression between the baseline and treatment phases (Tau-*U* = −0.829, *p* = 0.005). During the baseline phase, Samuel’s scores were all in the clinical range (“mild” to “severe”). There was a noticeable reduction in depressive symptoms after the initial treatment session (“telling my story”). Throughout the treatment and maintenance phases, his depression scores fluctuated between the “normal” and “mild” range.

No significant trend was found in the GAD-7 anxiety scores during the baseline phase (Tau-*U* = 0.048, *p* = 0.881). Phase comparison indicated a significant reduction in anxiety between the baseline and treatment phases (Tau-*U* = −0.7, *p* = 0.002). Samuel’s baseline scores were mainly in the “mild” to “moderate” range, although there was a notable reduction in the week prior to the treatment commencing. His scores fluctuated between the “normal” and “moderate” range during the treatment phase. During the maintenance phase, his levels of anxiety were in the “normal” to “mild” range.

On the ICQ, there was no significant trend found in the baseline phase for Samuel’s level of helplessness (Tau-*U* = −0.429, *p* = 0.177), acceptance (Tau-*U* = 0.095, *p* = 0.764), or perception of benefit (Tau-*U* = −0.333, *p* = 0.293). Phase comparison for helplessness indicated a significant decrease in levels of helplessness between the baseline and treatment phases (Tau-*U* = −0.771, *p* = 0.008). There was no significant difference between baseline and treatment phases for acceptance and perceived benefits (Tau-*U* = 0.486, *p* = 0.097; Tau-*U* = 0.086, *p* = 0.770).

Samuel’s FACT-G scores declined between the initial and final baseline assessments (see Table 7). His final baseline FACT-G score indicated low quality of life relative to the norms. On completion of the program, Samuel’s FACT-G scores had increased to within the range of 80–82, thus suggesting improvement in quality of life. Samuel’s scores indicated slight improvements in self-concept (i.e., increased continuity of self, continuity with others, and decreased discontinuity of self).

**Table 7:** **Broader subjective well-being scores for Samuel across phases**.

Measure	Initial baseline (A)	Final baseline (A)	End of treatment (B)	End of maintenance (C)
FACT-Brain
Physical	23	18	21	27
Social/family	21	21	21	19
Emotional	15	12	16	15
Functional	20	20	22	21
G	79	71	80	82
Brain	39	33	36	26
CDSS
Discontinuity self	28	26	24	26
Continuity self	12	13	13	14
Continuity others	19	16	21	21

Samuel reported high levels of therapeutic alliance on the SRS. With the exception of session six (where relationship and approach or method were rated at a score of 7), Samuel rated the alliance at 8 or higher [relationship (*M* = 9, SD = 0.94), goals and topics (*M* = 9, SD = 0.67), approach or method (*M* = 8.9, SD = 0.88), and overall (*M* = 8.9, SD = 0.57)].

In summary, visual and statistical analysis of Samuel’s psychological functioning indicated a clear reduction in his levels of anxiety, depression, and helplessness. Samuel’s levels of acceptance remained stable across the program while his perception of benefits was variable. There were minor improvements in his self-concept and quality of life.

## Discussion

The present study aimed to assess the feasibility and utility of a telephone-based psychological intervention for individuals with brain tumor. The evaluation of a telephone-based program was considered an important extension of the FTF MSoBT program (37) to provide a potentially more cost-effective option for the delivery of psychological support services, particularly for people living outside a major metropolitan area.

As the first study to evaluate a telephone-based psychological intervention for people with brain tumor, the SCED methodology provided a rigorous analysis of both within phase (i.e., baseline) and across phase variability on measures of psychological functioning. All four participants completed the intervention, which supports the feasibility of tele-based therapy for this population. Overall, two of the four participants demonstrated significant gains in mental health and more positive cognitive appraisals (John and Samuel). The other two participants (Mark and Robyn) did not demonstrate the hypothesized gains in mental health or cognitive appraisals. Despite these mixed findings, all participants reported some degree of improvement in quality of life and high levels of therapeutic alliance.

The mixed findings in the current research are consistent with those of previous support interventions that measured changes in psychological functioning. For example, Locke and colleagues (21) found that although participants increased their use of compensatory strategies, no significant differences in mood were found at post-intervention. However, unexpectedly, both Mark and Robyn reported an increase in mood symptoms over the course of treatment and lower levels of acceptance of the brain tumor. It is possible that discussion of recent and past stressors (e.g., marriage breakdown) heightened their awareness of the implications of their illness.

It is noteworthy that both Mark and Robyn had tumors located in the hypothalamic and pituitary areas. Mark identified difficulties with anger outbursts and emotion regulation and Robyn reported a major change in her emotional experiences prior to diagnosis (emotional blunting) and after diagnosis (mood swings and heightened emotions). Numerous studies have identified that dysregulation of the hypothalamic–pituitary adrenal axis contributes to mood disorders and difficulties down-regulating heightened emotional response to negative stimuli (64, 65). As a result, Mark and Robyn’s ability to apply the emotion regulation strategies (e.g., cognitive reappraisal) taught during the program in everyday situations may have been compromised by the nature of their brain injury.

Additionally, both Robyn and Mark identified cognitive difficulties that may have reduced the efficacy of telephone-based support. Mark displayed impaired verbal fluency which may have impeded his ability to express himself OTT, without the benefit of non-verbal cues. Robyn’s delayed verbal memory impairment may have affected her retention of the content of therapy sessions. Indeed, she identified in the latter part of the program that she is a visual learner and would have preferred FTF contact with the therapist. Further, Robyn and Mark both perceived a lack of understanding and support from friends, family, and colleagues. Therefore, despite a relatively favorable prognosis (i.e., Mark’s long-term survival with no re-growth or progression; complete removal of Robyn’s benign tumor), they both reported significant levels of emotional distress. This is consistent with previous research indicating that tumor characteristics are not consistently related to quality of life or psychological adjustment (3).

Further to this, impression management and insight were not measured throughout the program. Hence, it is possible that Mark was initially reluctant to fully disclose the extent of his distress during the baseline phase, and became more open about his mood symptoms and illness appraisals during the treatment phase. The use of self-report methods relies on the assumption that a participant will describe their symptoms and behaviors openly and accurately (66, 67).

In contrast to Mark and Robyn, both John and Samuel identified strong support systems through their family and social networks. Their stress associated with family and friends related to concerns around the loss of their independence and being a burden on the people they cared about. They also identified supportive workplaces where their roles were adapted to accommodate their difficulties, thus increasing their sense of life continuity (19). Psychotherapy techniques assisted with strengthening the roles identified as important to their sense of identity, despite their less favorable prognoses and ongoing health and functional difficulties. The tele-based therapy program provided a confidential space to discuss their hopes, fears, and concerns, without burdening or upsetting friends and family members.

### Clinical implications

Overall, results of this study provide some preliminary support for the feasibility and utility of telephone-based psychological support for people with brain tumor. Tele-based therapy may increase the opportunity to provide interventions to people otherwise unable to access brain tumor specific support. In the current study, a participant living over 4 h away from a major metropolitan area was able to be involved and he experienced significant gains in his psychological functioning. The tele-based intervention avoided the need for travel for all participants and the therapist, thus reducing common barriers to attending regular psychotherapy sessions, including transport, cost, and health-related barriers. As such, tele-based therapy may provide a cost and time-effective intervention option for individuals unable to access traditional (FTF) psychological support (39).

Despite the lack of FTF interaction, high levels of therapeutic alliance were reported by all four participants, suggesting that a strong rapport and alliance can be established OTT. As previously noted, therapeutic alliance is a strong predictor of outcome and thus the ability to establish good alliance on the telephone supports the viability of this therapy mode (41).

Participant feedback on intervention provided further important information about feasibility and utility of tele-therapy. As previously noted, Robyn stated that her first choice would have been for FTF contact due to her preference for visual processing of information. In particular, she found it disconcerting to only hear the therapist’s voice. Robyn regularly sent the therapist photos or art work via traditional mail (e.g., of a work function, of the table she sat at during therapy) in an effort to communicate using visual means. Robyn’s preference for FTF contact is consistent with research findings that 27.8% of participants had a preference for FTF contact, although this was not associated with treatment adherence (41).

In contrast, Mark and Samuel expressed surprise that rapport was so easily established without FTF interaction. Samuel also discussed the benefits of being able to undertake sessions from his own home, rather than having to travel which was difficult and upsetting for him due to the loss of his driver’s license. John expressed gratitude at being able to access psychotherapy, despite living outside a major metropolitan area. Like Samuel, he appreciated attending sessions from his own home to avoid fatigue. Overall, participants’ feedback indicated the importance of exploring people’s preferences for mode of delivery to enhance their experience of treatment, especially in the context of cognitive deficits.

Undertaking psychotherapy via telephone poses a number of unique challenges. To provide educational handouts, the practitioner needs to send material via traditional mail or email, prior to or after a session. The relevance of such materials is not always known in advance. There is a strong reliance on verbal cues (e.g., tone of voice) and feedback from the client regarding their understanding of the information and skills being trained. As such, there is the potential for increased preparation time for the therapist either prior to or after therapy sessions. This is particularly the case for clients with cognitive difficulties who often benefit from session summaries and visual aids (e.g., drawing a diagram to explain concepts) to process and retain new information (11). Further research is needed to explore the feasibility and utility of other tele-health or internet-based interventions (e.g., Skype and video-conferencing).

### Methodological considerations

The current SCED utilized a multiple-baseline design, examining stability of psychological functioning prior to treatment (51). Multiple baselines across participants and weekly observations over an extended period helped to assess for potential extraneous and confounding factors (49, 50). The combined use of statistical and visual analyses also increased the methodological rigor of the study, as recommended by recent methodological guidelines (50).

Despite these strengths, it is important to acknowledge potential limitations to generalizability. In particular, convenience sampling was used to recruit the four participants who were all proactive in help-seeking. Such characteristics may have enhanced their continued participation in the program and responsiveness to the intervention. As such, larger scale research (such as an RCT) should attempt to broaden intake processes to increase the representativeness of participants. Further, while each questionnaire administered has been validated in a brain injury, cancer, or community population, the measurement of change was based on self-report alone, with no collateral information obtained (48). As such, there is the potential for socially desirable responses, especially on measures of rapport and therapeutic alliance. Future research should attempt to measure outcomes independent from therapy, by use of a blind assessor or technology (i.e., online questionnaires), to reduce this potential (50). In addition, future research should attempt to obtain collateral information from a significant other or more objective indicators of psychological change (e.g., behavioral indices).

As the focus of the current research was on feasibility and utility, only a 5-week maintenance period was used. An extended period of follow-up (i.e., 3- or 6-month follow-up) is needed to assess more long-term benefits of telephone-based support interventions. Finally, future research may also benefit from assessing participants’ preference for intervention style (e.g., FTF or telephone-based). Whilst treatment adherence has not been found to be linked to preference (41), the outcomes of the intervention may have been influenced by the preference for FTF therapy. More generally, the circumstances in which tele-based psychotherapy is most effective for people with brain tumor need to be better understood.

## Summary

Overall, this study provides some preliminary support concerning the feasibility, practical benefits, and utility of tele-based psychological interventions for people with brain tumor. As the current study utilized four single-case studies, the ability for the findings to be generalized to the broader brain tumor population is limited. Nonetheless, the in-depth description of each participant and their intervention outcomes may enable clinicians to determine the relevance of the findings for their own setting. The results of this pilot study may guide future research on accessible and effective psychotherapy interventions for people with brain tumor.

## Author Contributions

All authors made a substantial contribution to the conception and design of the study, participant recruitment, data collection, and/or data analysis phases. Each author was involved in drafting the work or critically revising it for important intellectual content and gave final approval of the version to be published. All authors agree to be accountable for all aspects of the work in ensuring that questions related to the accuracy or integrity of any part of the work are appropriately investigated and resolved.

## Conflict of Interest Statement

The authors declare that the research was conducted in the absence of any commercial or financial relationships that could be construed as a potential conflict of interest.
